# Remote Sensing-Driven Climatic/Environmental Variables for Modelling Malaria Transmission in Sub-Saharan Africa

**DOI:** 10.3390/ijerph13060584

**Published:** 2016-06-14

**Authors:** Osadolor Ebhuoma, Michael Gebreslasie

**Affiliations:** School of Agricultural, Earth and Environmental Sciences, University of KwaZulu-Natal, Westville Campus, Durban 4000, South Africa; gebreslasie@ukzn.ac.za

**Keywords:** remote sensing, climatic/environmental variables, predictors, epidemiology, Sub-Saharan Africa

## Abstract

Malaria is a serious public health threat in Sub-Saharan Africa (SSA), and its transmission risk varies geographically. Modelling its geographic characteristics is essential for identifying the spatial and temporal risk of malaria transmission. Remote sensing (RS) has been serving as an important tool in providing and assessing a variety of potential climatic/environmental malaria transmission variables in diverse areas. This review focuses on the utilization of RS-driven climatic/environmental variables in determining malaria transmission in SSA. A systematic search on Google Scholar and the Institute for Scientific Information (ISI) Web of Knowledge^SM^ databases (PubMed, Web of Science and ScienceDirect) was carried out. We identified thirty-five peer-reviewed articles that studied the relationship between remotely-sensed climatic variable(s) and malaria epidemiological data in the SSA sub-regions. The relationship between malaria disease and different climatic/environmental proxies was examined using different statistical methods. Across the SSA sub-region, the normalized difference vegetation index (NDVI) derived from either the National Oceanic and Atmospheric Administration (NOAA) Advanced Very High Resolution Radiometer (AVHRR) or Moderate-resolution Imaging Spectrometer (MODIS) satellite sensors was most frequently returned as a statistically-significant variable to model both spatial and temporal malaria transmission. Furthermore, generalized linear models (linear regression, logistic regression and Poisson regression) were the most frequently-employed methods of statistical analysis in determining malaria transmission predictors in East, Southern and West Africa. By contrast, multivariate analysis was used in Central Africa. We stress that the utilization of RS in determining reliable malaria transmission predictors and climatic/environmental monitoring variables would require a tailored approach that will have cognizance of the geographical/climatic setting, the stage of malaria elimination continuum, the characteristics of the RS variables and the analytical approach, which in turn, would support the channeling of intervention resources sustainably.

## 1. Introduction

Malaria remains the number one killer of all infectious diseases in Sub-Saharan Africa (SSA) [1]. In 2013, an estimated 198 million malaria cases and 584,000 malaria deaths were recorded. About 90% of the malaria deaths recorded were from the SSA region [2]. Out of all known malaria parasites, viz. *Plasmodium falciparum*, *P. vivax*, *P. ovale*, *P. malariae* and *P. knowlesi* [2], *P. falciparum* is the most prevalent of the human malaria parasites in SSA, while the *P. vivax* malaria parasite is more common across the Horn of Africa [3]. The spatial and temporal variation of malaria disease is known to be influenced by socio-economic/human, ecological/environmental and climatic factors [4,5]. The climatic variables suggested to possess a direct and indirect influence on malaria transmission are rainfall, temperature, altitude and humidity [6,7,8,9,10,11,12]. Rainfall expands mosquito breeding habitats, which in turn, increases its population densities and risk of malaria transmission [13]. Nevertheless, excessive rainfall is also capable of flushing the breeding sites [14]. The completion of the malaria life cycle is suited to temperatures between 15 °C and 40 °C and between 16 °C and 33 °C for the development and survival of mosquitoes [13,15]. Regarding bite rates and feeding habits, at 17 °C, mosquitoes take a human blood meal every four days, while at 25 °C, they feed on humans every two days [13]. Altitude has an indirect relationship with temperature, and as such, areas above 1500 m in Africa have little or no risk of malaria transmission [16]. Relative humidity above 60% does not substantially affect the longevity of mosquitoes, but relative humidity lower than 10% results in death within hours [17], while malaria parasites develop between 55% and 80% humidity [18]. The aforementioned climatic variables have been shown to be important malaria transmission indicators that can be used to determine and predict the spatial and temporal distribution of the disease. Consequently, this can guide malaria control managers in decision and policy making in distributing cost-effective intervention resources in time and space [19]. 

There has been a wide interest in the development of reliable malaria risk maps, forecast models or integrated malaria early warning systems based on the combination of historical malaria case data and selected climatic/environmental variables [6,8,9,10,11,12,20] so that intervention resources can be channeled before an epidemic occurs [19]. This further indicates the importance of acquiring and utilizing historic climatic variables to adequately study and understand the role they play in the temporal and spatial heterogeneity of malaria. Historical climatic variables obtained from meteorological stations have proven to be vital in identifying and modelling malaria transmission [21]. However, limited numbers of meteorological stations, which are additionally located far apart, and malfunctioning of meteorological station resources make it challenging to obtain historical and spatially-continuous observations of climatic/environmental variables on a wider geographical scale in SSA [22]. Therefore, the need to search for and acquire alternative, indirect or proxy data from remote sensing (RS) is essential [20,22]. 

The emergence of RS satellites provided a wide array of environmental variables at different spatial and temporal scales [23,24,25,26,27,28], which then created an avenue to increase our understanding of the association between malaria disease and a variety of environmental/climatic variables [4,29,30]. Detailed assessment, evaluation and understanding of this technology in relation to malaria is needed to adequately harness its potential, which in turn, would enhance spatial risk modelling and identification of reliable malaria transmission predictors. Therefore, the aim of this review is to appraise the utilization and applications of RS technology and to discuss its contribution in enhancing the understanding of malaria transmission dynamics in SSA with a focus on RS-driven climatic/environmental variables. This paper will serve as a framework for health practitioners and researchers aiming to identify relevant climatic/environmental variables that are highly related to malaria in particular localities and regions in SSA. 

The paper is organized in the following main sections. Initially, we described how the reviewed articles were searched and retrieved. Secondly, we illustrated the malaria risk stratification of the study region, and thirdly, we summarized studies by region (East, Southern, West and Central Africa) that used RS-derived variables in modelling malaria transmission and risk. Finally, we discussed the relevance and implication of the RS-derived variables peculiar to regions and the possible rationale behind their usage. Furthermore, we suggested ways in which the usage of RS-derived variables can be maximized in determining reliable malaria transmission predictors.

## 2. Materials and Methods 

### 2.1. Search Strategy

**Figure 1:**
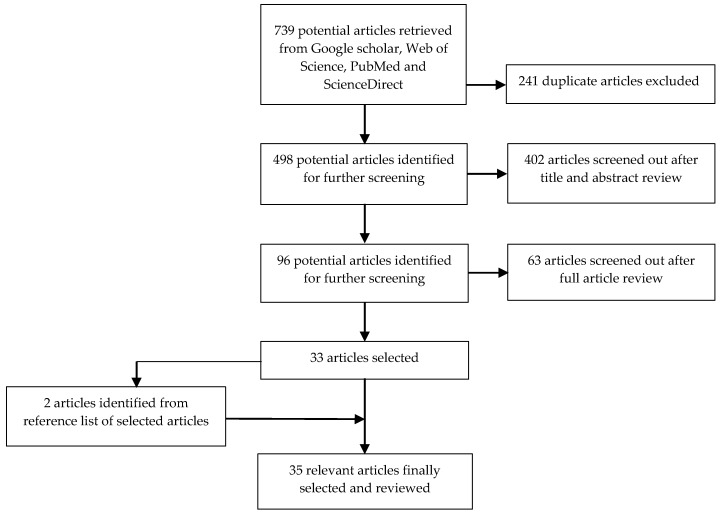
Flow chart of publication screening and selection processes.

A systematic search to retrieve relevant literature and referenced articles began in September 2014, and the final search was conducted in March 2015. The search was aimed at identifying epidemiological studies in SSA that utilized RS-derived climatic/environmental variables in mapping, modeling or forecasting malaria by carrying out a search on Google Scholar and the ISI Web of Knowledge^SM^ databases: PubMed, Web of Science and ScienceDirect. The database queries were formulated using Boolean operators to combine two or more keywords. The keywords were identified and selected from public and environmental health studies, epidemiological studies and subject headings. The keywords were “remote sensing”, “geographical information system”, “Earth observation”, “spatial techniques”, “geo-spatial analysis”, “geo-spatial techniques”, “malaria”, “forecasting”, “modelling”, “mapping”, “prediction”, “epidemic”, “climate change”, “climatic factors”, “climatic variables”, “environmental proxies”, “temperature”, “rainfall”, “normalized difference vegetation index (NDVI)”, “humidity”, “EL Nino Southern Oscillation”, “West Africa”, “Central Africa”, “East Africa”, “Southern Africa” and “Sub-Saharan Africa”. Titles and abstracts were initially examined to determine their relevance. Thereafter, the full texts were downloaded to ascertain if they met the selection criteria listed below. Finally, the reference list of each relevant article was assessed to identify other relevant article(s). The search strategy, screening and selection processes are illustrated in Figure 1.

### 2.2. Selection Criteria

The selection criteria involved *post hoc* inclusion and exclusion criteria suggested by Arksey and O’Malley [31] and Levac *et al.* [32]. They were developed based on familiarity with the subject matter through reading articles and reviews around malaria epidemiology. The authors discussed and agreed on the study inclusion and exclusion criteria at the beginning of the selection process, and various stages of the conceptual review stages and the selection criteria were refined until the final selection criteria were accepted. This enables us to eliminate studies that were outside the scope of our study aim and ensured consistency.

The articles finally selected were:

1. Original peer-reviewed articles published in English between 1 January 2000 and 31 December 2014. The search period was selected because since 2000, robust appreciation and application of RS in malaria studies occurred, which can be attributed to the easy access of RS data and the emergence of improved remote sensing sensors. Furthermore, this period coincides with the availability of MODIS data [33].
2. Articles that applied RS-derived climatic/environmental variables and/or climatic proxy indicators in evaluating malaria risk, distribution, transmission and mapping.
3. Studies that assessed the impact of inter-annual climate variability on malaria transmission. Studies in which climate change projections were used to estimate future malaria distribution were excluded.
4. Publications that used malaria incidence and/or prevalence data in their epidemiological study design (descriptive/explorative, spatial and/or temporal analysis and time series analysis). Studies that used only entomological data were excluded.
5. Studies conducted in Sub-Saharan Africa. Continental-wide studies were excluded because many African countries have made significant progress in fighting malaria, and malaria is clustered in small areas.

### 2.3. Description of the Study Region

**Figure 2:**
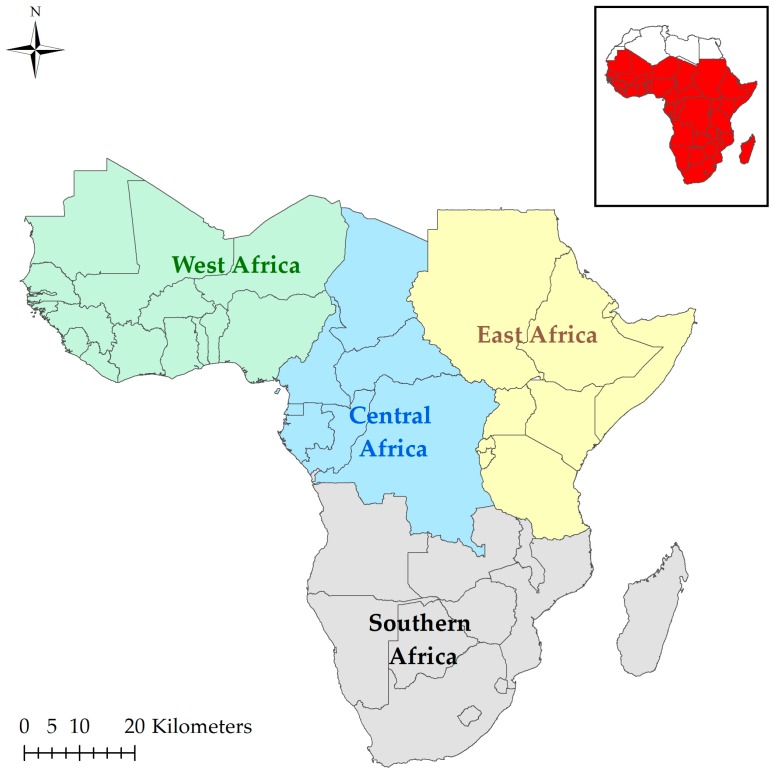
Map of SSA showing the study regions.

**Figure 3:**
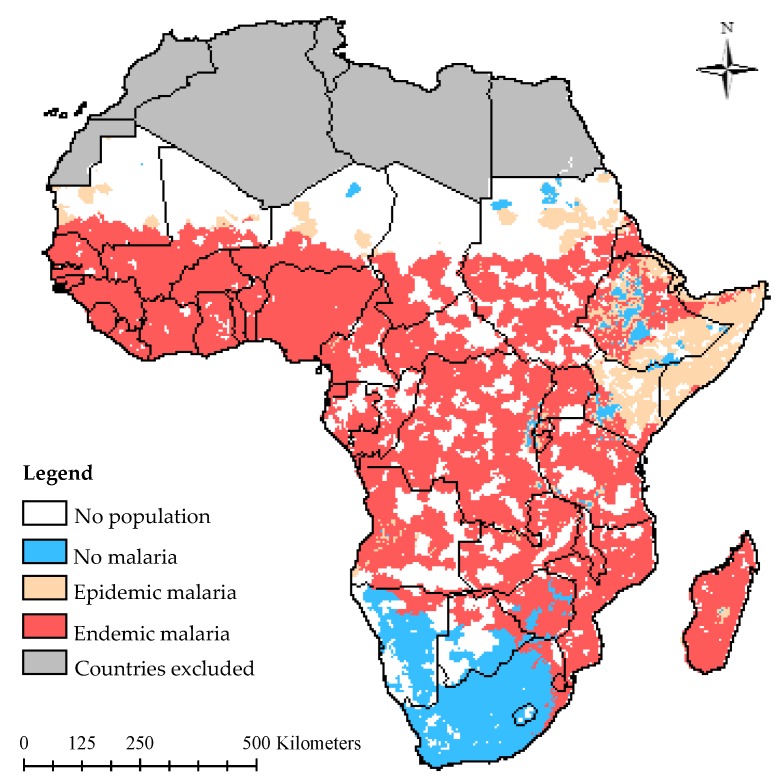
Malaria risk stratification of SSA (MARA/ARMA [34]).

SSA can be sub-divided into four regions (East, West, Central and Southern Africa) as shown in Figure 2. Malaria is endemic in a substantial part of SSA where the climate supports 20%–100% suitability (Figure 3) [34]. At the fringes of this region, there are areas where malaria rarely occurs because the climate is not always suitable. Nevertheless, variation in weather or climatic conditions could instigate an epidemic. The changes in climatic conditions are normally due to higher than normal rainfall and temperature in desert and highland fringes, respectively [34,35]. 

Malaria stratification in East Africa varies notably and can be attributed to the varying and complex climate associated with the region [36]. The climate of the endemic parts of East Africa favors a seasonal and perennial transmission of malaria, while in substantial parts of Kenya and parts of the Horn of Africa, malaria transmission is strongly seasonally prone to epidemics (1–3 months) owing to low rainfall and inter-annual variability [34]. The White Nile River, Blue Nile River, Lake Victoria, Lake Albert, Lake Tanganyika and Lake Malawi, coupled with the varying climate, are significant risk factors for endemic and epidemic malaria in the region. 

Monthly temperature variations, which peak in the rainy season [37], coupled with rainfall that increases towards the Equator, may be responsible for the highly seasonal and varied malaria suitability in Southern Africa [36]. The climate in the malaria-free areas does not totally support malaria endemicity. However, environmental factors, such as the Orange River, which runs through Lesotho, Namibia and South Africa, and the Zambezi River in Botswana, can potentially support malaria epidemicity, while the parts of Southern Africa endemic to malaria are supported by suitable climate and water bodies (Limpopo and Zambezi Rivers) that favor seasonal malaria transmission [34]. 

The endemicity of malaria spans across West Africa substantially, excluding only the desert and semi-desert areas. The region is characterized by the Sahelian, Sudanian, tropical humid and equatorial climates. In this region, temperature increases northwards while rainfall increases southwards [36]. The region supports seasonal (4–6 months) and perennial (7–12 months) malaria transmission [34]. In addition, major water bodies like rivers (Benue, Niger, Volta and Senegal) and lakes (Volta and Chad) can sustain malaria transmission in the region.

Central Africa is significantly endemic to malaria. Suitable climatic conditions (relatively high and reliable rainfall over the coastal and central parts and a temperature range between 19 °C and 28 °C) [36] coupled with the Congo River, Lake Tanganyika and Lake Albert, contributes to the perennial transmission of malaria experienced in the region [34]. 

## 3. Results

Initially, 739 related publications were identified. After going through them thoroughly and carefully according to the search strategy and selection criteria, 35 articles were finally selected. From the selected articles, most of the study sites were located in East Africa (14 studies; Table 1), followed by Southern Africa (11 studies; Table 2) and then West Africa (nine studies; Table 3). The only study that covered the Central Africa region utilized datasets covering both Central and West Africa (Table 4). The study area(s), malaria case data, climatic variables and their sources, the statistical methods used and the main findings are provided for each study in Table 1, Table 2, Table 3 and Table 4, while Table 5 provides an overview of the RS variables commonly used in SSA, and Table 6 provides the characteristics of the satellites/sensors used in the selected articles.

### 3.1. East Africa

In East Africa, country-specific studies took place mainly in Kenya (four studies), Eritrea (two studies) and Somalia (two studies). Other study locations were Ethiopia, Burundi and Tanzania. Three cross-national studies were identified. One study used data that cuts across Kenya, Ethiopia and Uganda [38], while two other studies used national data from Kenya, Uganda and Tanzania [39,40] (Table 1). The East African countries identified in Table 1 are currently in the control phase of the World Health Organisation (WHO) malaria elimination continuum [41]. Studies conducted in the region mainly used National Oceanic and Atmospheric Administration (NOAA) Advanced Very High Resolution Radiometer (AVHRR) imagery as a source of proxy climatic/environmental variables for modelling malaria transmission both at the country and cross-national level. NDVI was observed to be the most assessed RS-derived variable and also the most statistically-significant malaria transmission predictor across East Africa. In the province of Karuzi in Burundi, Gomez-Elipe *et al.* [42] used NDVI extracted from NOAA-AVHRR at an 8 km × 8 km spatial resolution, while rainfall and maximum and minimum temperatures were obtained from the metrological stations. After employing the autoregressive integrated moving average (ARIMA) model, NDVI, rainfall and maximum temperature were observed to correlate with malaria cases, and hence, it constituted the best predicting model (*R*^2^_adj_ = 82%, *p* < 0.0001 and 93% predicting accuracy). Ceccato *et al.* [43] used Spearman’s and Pearson’s rank correlations to assess the relationship between malaria incidence and climate/environmental variables anomalies (to eliminate the similar seasonal pattern possessed by both dependent and independent variables) in Eritrea. The climatic/environmental variables used by these authors included NDVI from NOAA-AVHRR at 8 km × 8 km spatial resolution, rainfall estimates (RFE) from Climate Prediction Centre Merged Analysis of Precipitation (CMAP) at a 2.5° × 2.5° grid and rainfall data from metrological stations.

NDVI anomalies were highly correlated with malaria incidence anomalies, particularly in the semi-arid north of the country and along the northern Red Sea coast, which is a highly epidemic-prone area. CMAP rainfall correlated with malaria incidence anomalies, with a lead time of 2–3 months, while weather station rainfall correlated with malaria anomalies with a delay of two months. Generally, the correlation coefficients were between 0.6 and 0.8. Similarly, Graves *et al.* [25] analyzed the effects of impregnated nets, larval control, malathion and DDT on malaria cases, while analyzing the effects of RS-derived climate variables, such as NOAA-AVHRR NDVI (8 km × 8 km spatial resolution) and CMAP RFE (2.5° × 2.5° grid) in Eritrea at the district level. The Poisson regression analysis employed showed that the relation between the climatic variables and malaria cases varied by zones. The increase in malaria cases was significantly associated with RFE with a lead time of 2–3 months (0.0007711, *p* < 0.001) in the Gash Barka zone and NDVI anomalies in the current and previous months (1.820668, *p* < 0.0001). NDVI also exhibited the same relationship in the Anseba zone, but with a greater coefficient (11.22517, *p* < 0.001). Gosoniu *et al.* [48] employed Bayesian geostatistical models to analyze the effects of parasitemia risk (malaria cases among children less than five years old) with age, socio-economic status (wealth index and residence), malaria intervention (bed nets) and climatic/environmental factors (Moderate Resolution Imaging Spectrometer (MODIS) land surface temperature (LST), MODIS NDVI, altitude from the United States Geological Service (USGS) digital elevation model (DEM), RFE data from the Meteosat-7 satellite obtained from the Africa Data Dissemination Service (ADDS) and distance to nearest water body obtained from Health Mapper) in Tanzania. The spatial resolution of the environmental/climatic factors used was 1 km × 1 km, except rainfall, which was 8 km × 8 km. Altitude was negatively associated with malaria risk at the 5% significance level, indicating that children living above 1500 m had a lower risk of malaria, while rainfall, NDVI and day and night LST were positively associated with parasitemia risk. In a study by Omumbo *et al.* [39], malariometric data and RS-derived variables (NDVI, mid-infrared (MIR) reflectance, cold cloud duration (CCD), land surface and air temperature indices and altitude) from Kenya, Uganda and Tanzania were used to update the spatial resolution of their malaria transmission risk map. These authors pre-processed the RS-derived data using the temporal Fourier analysis, and the discriminant analysis that was employed subsequently revealed that NOAA-AVHRR LST was the best predictor of malaria transmission intensity, while NOAA-AVHRR NDVI and CCD derived from the Meteosat satellite were identified as secondary predictors of transmission intensity. The forecast was significantly improved by altitude derived from USGS-DEM. Areas with moderated malaria were under-forecasted (false negative rate = 27.7%), while malaria-free areas were over-forecasted (false positive rate = 26.3%). In a similar study that used data covering Kenya, Uganda and Tanzania, Omumbo *et al.* [40] discovered that NDVI, CCD and water body area were associated with malaria in the “dry” Ecozone 1 (arid and highland, with a climate that favors few months of mosquito proliferation). In Ecozone 2 (diverse, with a climate that supports the propagation of mosquitoes for longer transmission seasons), temperature variables were identified as the most abundant variables in the prediction model. The addition of ecological zoning improved the overall model accuracy by 6.1%, and kappa values increased from 0.397–0.477.

### 3.2. Southern Africa

Studies in Southern Africa in which the relationship between RS-derived climatic variables and malaria incidence and/or prevalence were identified are summarized in Table 2. Study sites in Southern Africa mainly included Botswana (three studies), Zimbabwe (two studies) and Zambia (two studies). Other locations included Angola, Namibia, Swaziland and Malawi. Most Southern African countries, including Mozambique, Angola, Zimbabwe, Malawi and Zambia, are in the malaria control stage of the malaria elimination continuum [41]. South Africa, Botswana and Namibia are in the pre-elimination stage [41], while Swaziland is in the elimination stage [70]. RS-extracted climatic and environmental variables used in this region were obtained mainly from NOAA-AVHRR and MODIS satellite sensors. NDVI was observed to be the major RS-derived variable linked to malaria transmission in the region followed by RFE. Gosoniu *et al.* [28] fitted Bayesian geostatistical models to assess the effects of malaria intervention (insecticide-treated nets) among children less than five years old in Angola between 2006 and 2007 after adjusting for socio-economic status and climatic/environmental factors (MODIS LST, MODIS NDVI, altitude derived from USGS DEM, distance to nearest water body from Health Mapper and rainfall from ADDS). These authors examined the association between malaria incidence and climatic/environmental factors and found that NDVI (95% CI = 6.28, 17.94; OR = 10.62) and rainfall (95% CI = 6.00, 19.43; OR = 10.80) had a significantly positive relationship with malaria incidence. Similarly, Riedel *et al.* [55] investigated the relationship that existed between malaria interventions and malaria risk after adjusting for selected RS (MODIS LST, MODIS NDVI, MODIS land cover, ADDS RFE, altitude from USGS DEM, water bodies) and socio-economic variables in Zambia. A spatially independent and Bayesian geostatistical model was generated that used malaria cases from the Zambia Malaria Indicator Survey conducted in 2006. NDVI, night LST and rainfall (the last 2.7 months) were identified as positive significant predictors of malaria and were fitted in the model. Cohen *et al.* [57] conducted a study aimed at generating a case-based risk map for Swaziland using the 2011 malaria case data obtained from the Swaziland National Malaria Control Programme. Ecological variables, such as rainfall (from weather stations), temperature (from worldClim), NDVI, normalized difference water index (NDWI), elevation, topographic wetness index (TWI) and water bodies (from the Food and Agriculture Organisation of the United Nations) were assessed for their relevance in the formulation of a high spatial and temporal resolution malaria risk map. NDVI and NDWI data were calculated from a high spatial resolution imagery (30-m spatial resolution) from the Landsat 7 Enhanced Thematic Mapper plus (ETM+) sensor. The Landsat 7 ETM+ is an improvement of the previous Landsat satellite series that provides medium-resolution multispectral imagery of the Earth’s surface [33]. Elevation and TWI were obtained from the Shuttle Radar Topography Mission (SRTM) at 90-m spatial resolution. These authors suggested that during the high transmission season, malaria cases tend to cluster in areas of lower elevation, closer to water bodies, in less populated areas, with lower rainfall and lower temperatures (all *p* < 0.001). In relation to the model accuracy, NDWI was the most important RS-derived predictor followed by NDVI and TWI. Finally, models formulated from the random forest classification were used to produce predicted probability case-based maps. Nygren *et al.* [26] explored the relationship between RS-derived environmental malaria transmission and forecasted malaria cases in the Southern Province of Zambia. The RS-derived variables included MODIS NDVI, MODIS nocturnal dew point (DWP), MODIS LST, rainfall and elevation. The rainfall data were obtained from the Tropical Application of Meteorology using satellite data and ground-based observations (TAMSAT). TAMSAT rainfall data are rainfall estimates obtained from Meteosat (from the thermal infrared channels) and calibrated against rainfall data from rain gauges [71,72]. In addition, elevation data at 30-m spatial resolution was derived from the Advanced Spaceborne Thermal Emission and Reflection Radiometer (ASTER), which is a sensor on-board the Terra satellite for DEM creation [33]. NDVI, DWP and night LST were the highly significant predictors in the high and low malaria transmission areas, and the NDVI and DWP improved the ARIMAX models in all areas significantly. The mean average error of the forecast models was between 0.7% and 33.5%.

### 3.3. West Africa

In West Africa, country-specific studies took place mainly in Mali (four studies) and later Côte d’Ivoire (three studies). Others studies were conducted in Gambia and Senegal. One regional study used malariometric data obtained from Mapping Malaria Risk in Africa (MARA/ARMA), which covered West Africa, but excluded Cape Verde [6]. All of the West African countries are in the control stage of the WHO malaria elimination continuum, except Cape Verde, which is in the pre-elimination stage [41]. A summary of studies in West Africa that used RS climatic/environmental variables to identify climatic/environmental predictors of malaria is given in Table 3 above. In West Africa, the most frequently-utilized RS climatic/environmental variables were from NOAA-AVHRR and MODIS sensors. In the region, NDVI was identified as the major RS climatic predictor of malaria risk and transmission. Giardina *et al.* [63] used malaria prevalence data from Senegal’s Malaria Indicator Survey to determine spatially-explicit climatic/environmental variables associated with malaria in Senegal by incorporating Bayesian variable selection methods within a geostatistical framework. The formulated model included night LST (OR = 1.16; 95% CI (0.66, 1.86)), NDVI (OR = 1.48; 95% CI (0.88, 2.48)), urban area (OR = 0.19; 95% CI (0.07, 0.45)) and rural area (OR = 1), and they were noted to have a positive association with malaria parasitemia risk. Similarly, Gosoniu *et al.* [27] estimated the burden of malaria in Mali by using a Bayesian non-stationary model. Malaria prevalence data were extracted from the MARA/ARMA, 1998 database, NDVI from NASA-AVHRR, temperature and rainfall obtained from Hutchinson *et al.* [73], water bodies from World Resources Institute [74] and season length from Gemperli *et al.* [65]. The best sets of variables included in the non-stationary model were NDVI and minimum temperature, which had a positive significant relationship with malaria risk. Contrarily, rainfall had a negative significant relationship. The authors further suggest that stationarity assumptions are vital due to their influence on the significance of environmental parameters and parasitemia risk map. Gaudart *et al.* [23] incorporated RS-derived variables into a temporal model to predict malaria transmission in the locality of Bancoumana, Mali, characterized by Sudanese savannah. Confirmed *P. falciparum* data obtained from a field study of children aged 0–12 years and 15-day composites of NDVI data derived from NOAA-AVHRR between 1981 and 2006 were incorporated in the ARIMA time series. The analysis revealed that the seasonality of *P. falciparum* incidence was significantly explained by NDVI with a 15-day lag (*p* = 0.001), and the threshold was 0.361 (*p* = 0.007). The deterministic malaria transmission model, with stochastic environmental variables, forecasted an endemoepidemic pattern of malaria, and the value of the adjusted *R*^2^ was 89%. Similarly, in a study conducted by Kleinschmidt *et al.* [59], malaria risk was determined on a large scale by identifying important ecological parameters, and subsequently, a malaria risk map was produced for Mali. These authors used an automatic stepwise variable selection procedure to identify the most reliable predictors of malaria prevalence for the multiple logistic regression model. NDVI from June–November (wet season), mean maximum temperature from March–May, months with more than a 60-mm rainfall and distance to water bodies were the significant independent variables for predicting malaria prevalence and were incorporated into the final multiple logistic regression model; and finally, a map of malaria risk was formulated. On the other hand, Silue *et al.* [61] used the Bayesian model to produce spatially-explicit risk maps of malaria transmission in Man, Côte d’Ivoire. Initially, these authors analyzed the relationship of malaria prevalence data with possible malaria transmission risk factors, including age, use of bed nets, socio-economic status, distance to health facilities, NDVI, rainfall, LST and distance to rivers. NDVI and LST were extracted from MODIS at a 1 × 1 km spatial resolution, while RFE from the Meteosat-7 satellite was obtained from the ADDS at an 8 × 8 km spatial resolution. In bivariate non-spatial models, NDVI, RFE and distance to rivers were significantly associated with a *P. falciparum* infection. However, after employing the spatial correlation analysis, only age was noted to be a significant risk factor for malaria prevalence, while NDVI showed a “borderline” significance. 

### 3.4. Central Africa

In the Central African region, the only study we identified that examined the association of malaria with RS climatic and environmental characteristics is given in Table 4. This is a cross-regional study that used Malaria prevalence data obtained from the MARA/ARMA database and numerous malaria transmission factors, including population density, NDVI, land use, temperature, rainfall, water bodies, soil water storage index, agro-ecological zone and transmission seasonality covering Central and West Africa [65]. The authors discovered that NDVI extracted from the NASA-AVHRR sensor at an 8 × 8-km spatial resolution had a high relationship with malaria across the region, except in areas far away from water bodies. Furthermore, a negative association was recorded between malaria transmission and distance to water, and this was observed in regions with NDVI values greater than 0.6. The spatial and non-spatial variations were 0.398 and 41.98, respectively. With reference to the WHO malaria elimination continuum, all of the Central African countries are still in the control phase [41], excluding São Tomé and Príncipe, which are currently in the pre-elimination stage. 

### 3.5. Commonly-Used RS Variables and Features of Satellites/Sensors Used by the Authors in the Articles Reviewed

Table 5 provides an overview of the RS variables commonly used in SSA, while Table 6 presents the satellite sensors used in the various selected studies (these sensors have different spatial, spectral temporal, radiometric and swath width properties). NOAA-AVHRR and MODIS were the most frequently-utilized sources of RS-derived indices, such as NDVI, EVI, LST, ETa and DWP across SSA. In addition, Meteosat [26,39,40,61,62,75] and the Climate Prediction Centre Merged Analysis of Precipitation (CMAP) [25,43,51,53,58] were also used extensively to extract RFE and precipitation data. 

## 4. Discussion

Our review highlights the contribution of RS technology in modelling malaria transmission and risk in SSA after taking account of potential climatic/environmental variables that can be used to predict malaria transmission. Malaria disease exhibits seasonal and spatial heterogeneity across localities, districts, provinces, countries and also in sub-continental regions. This can be attributed to the complex nature of malaria resulting from the diverse climatic, environmental, social and natural elements supporting the disease. The combination of these factors plays an important role in the endemicity and epidemicity of an area. RS serves as a means of obtaining potential climatic/environmental malaria variables and opens an avenue to better understand and model the environmental/climatic processes fundamentally responsible for the temporal and spatial heterogeneity of malaria disease.

RS has proven to be a vital tool in malaria modelling and prediction. It can contribute to malaria intervention planning and control programs at both local and broad scales and at different malaria risk stratifications. The scarcity of reliable meteorological data, national health policies and priorities, research/institutional capacity, availability and the cost of high resolution RS data for research and public health purposes determines the use of RS in malaria modelling [76]. This notwithstanding, RS-derived variables are gaining widespread acceptance and application in malaria risk modelling in SSA because of the nature and characteristics of a variable of interest can reflect the ecological relevance and contribution to malaria transmission. For instance, RFE provides indirect estimates of rainfall based on the detection of precipitation particles or the duration a cloud top is below a threshold temperature [69]. LST can be used as a proxy for temperature, and its values are obtained from land surface emissivity or surface reflectance in relation to their wavelengths and spectral characteristics [68], while NDVI can serve as a surrogate for rainfall, temperature, land use and land cover, near-surface humidity and surface water [20,77]. Thus, RS-derived variables have the potential to provide information that directly exhibits the state of the vector habitat and to inform us about the potential role that ecology can play in malaria transmission [29,68].

The robust utilization of RS-derived variables across SSA has shown that malaria predictors and models are peculiar and subject to the influence of the reference data, scale of observation and environmental condition of the study area. For example, in sub-continental East Africa, we observed that NDVI extracted from NOAA-AVHRR at 8 km × 8 km and MODIS at 1 km × 1 km spatial resolutions is an important predictor of malaria transmission at the country level in Kenya, Tanzania [48], Burundi [42] and Eritrea [25,43]. However, at the local level, in the rich herbaceous and cropland vegetation of the Amhara region, which constitutes the Ethiopian Highlands, NDVI obtained from MODIS at a 1 km × 1 km spatial resolution was not significantly related to malaria. Instead, ETa (which was only recently assessed for its relevance in malaria risk profiling), EVI and LST variables extracted from MODIS at a 1 km × 1 km spatial resolution were observed to be the suitable malaria predictors in Amhara, Ethiopia [20]. This can be explained by the fact that NDVI loses sensitivity in areas of higher vegetation density and at higher EVI values. The vegetation index EVI can be used as a substitute for NDVI, because it preserves more sensitivity over heavier vegetation; hence, good account of the variation and the change in a rich canopy can be recorded [78,79]. However, the application of EVI in malaria risk profiling and modelling was used sparingly in the East African sub-region and other SSA regions.

The pronounced climatic diversity in relation to malaria suitability at the country level in Southern Africa may have contributed to the diverse RS variables identified as significant malaria predictors in the sub-region. However, NDVI extracted from MODIS at 1 km × 1 km [26,28] and 0.25 km × 0.25 km [55] and Landsat 7 ETM+ at 30 m × 30 m spatial resolutions [57] can be used to explain the geographical spread of malaria in greater parts of the malaria endemic areas when compared to other RS-derived variables identified as significant predictors of transmission. On the other hand, in the malaria endemic region of Northern Namibia, Alegana *et al.* [56] found that MODIS EVI at a 1 km × 1 km spatial resolution and precipitation derived from TRMM at a 0.25° × 0.25° spatial resolution, which was re-sampled to a 1 km × 1 km spatial resolution, were the best malaria predictors, but it must be noted that NDVI was not considered in the study. 

NDVI continued to exhibit its dominance in usage and significance pertaining to malaria risk determination across the SSA regions. NDVI extracted from either NOAA-AVHRR at an 8 km × 8 km spatial resolution or MODIS at a 1 km × 1 km spatial resolution, respectively, was identified as a suitable malaria predictor in Western Africa, especially in settings characterized by the Sahelian or Sudanian climate at the local level (Bancoumana, Mali) [23] and the country level (Mali and Senegal) [27,59,63]. However, in areas characterized by persistent moisture and heavy vegetation, different outcomes were observed. In the Man region of Côte d’Ivoire, Raso *et al.* [62] identified RFE data obtained from the Meteosat 7 satellite at an 8 km × 8 km spatial resolution as the predictor for malaria prevalence. Furthermore, a significant negative association between plasmodium prevalence and MODIS LST at a 1 km × 1 km spatial resolution was recorded in a study that used data covering Cote d’Ivoire [64].

In the Central African region (characterized by climatic suitability for malaria proliferation and heavy vegetation), the only study we identified and reviewed indicated that NDVI calculated from the NASA-AVHRR sensor at an 8 × 8 km spatial resolution returned a better result for modelling malaria transmission [65]. EVI, which has been suggested to be an alternative predictor over denser vegetation than NDVI, may have been identified as a better malaria predictor in the Central African region, but it was not included in the study. Furthermore, other climatic/environmental factors used in the study may have been identified as suitable malaria predictors, but the authors did not consider the differences that might exist in the climate-malaria relationship across the study area. In addition, they did not take into account the non-stationary characteristics of malaria data covering large areas. Disregarding this characteristic could result in the wrong specification of the spatial correlation and, therefore, erroneous values of the standard error of the predictors and prediction. In a somewhat similar study conducted by Gosoniu *et al.* [6], the authors used data covering West Africa and addressed the above-mentioned issues by partitioning the study area into four agro-ecological zones and then employed a different non-parametric model in each zone. There is a possibility that more studies in the Central African region may be available, but could not be identified, as the region is dominated by French-speaking countries. Hence, researchers from the region may have their articles published in French.

The tremendous improvements in the RS sensors, better turnaround time and availability of some RS low and medium resolution datasets at no cost [80,81] may have also contributed to the considerable utilization of various RS datasets across SSA. The freely available RS datasets obtainable via MODIS and AVHRR satellites can be used to explain the frequent usage of these satellites as compared to RS datasets from other RS sources [33,82]. Furthermore, MODIS has made it possible to evaluate new and previously unidentified environmental-related malaria predictors. For example, contemporary studies have shown that MODIS DWP at a 5 km × 5 km spatial resolution and MODIS ETa at a 1 km × 1 km spatial resolution can be used to explain and define malaria transmission risk and malaria incidence in the Southern Province of Zambia and the Amhara region of Ethiopia, respectively. 

Countries in SSA are at different stages in their fight towards malaria elimination, and this has to be taken into account in line with the characteristics of the RS imagery intended to be used. Low and medium spatial resolution RS data can be useful in studies conducted at national and regional levels in the malaria endemic countries that are still at their malaria control stage; in addition, to derive generalized spatio-temporal models and a malaria risk map for the robust application of intervention resources. However, in Angola, Botswana, Cape Verde, Namibia, Swaziland and South Africa with significantly low malaria cases [41], high spatial resolution RS data at a local level would be essential to carry out the cluster analysis and detection of foci and hotspots of malaria transmission. This would support adequate monitoring of the disease and delivery of interventions to specific location(s) and/or seasons, ultimately leading to malaria reduction. New generation satellites, such as Landsat-8, Copernicus: Sentinel-2, the Global Precipitation Measurement (GPM) mission, the Soil Moisture Active/Passive (SMAP) mission, SPOT 6 and SPOT 7 [33], with improved spatial and radiometric resolutions, have potential for malaria transmission and risk modelling, especially in regions where malaria cases are low (Table 7). Furthermore, future satellite mission, like Copernicus: Sentinel-3, which would introduce data reliability for long-term monitoring, could also be vital in modelling spatial and temporal malaria transmission and research [33]. The cost of obtaining high spatial resolution datasets remains a challenge in SSA. Hence, lessons can be drawn from the collaborative venture that exists between China and Brazil, which allows their researchers to obtain high spatial resolution data (2.5 m) freely [83].

In a bid to identify relevant and potential risk factor(s) or malaria transmission predictors at local, national or regional levels that can be further incorporated into forecast models/early warning systems and malaria risk maps, the statistical methodology employed should accommodate procedures that suit a particular context and setting. According to Table 1, Table 2, Table 3 and Table 4, generalized linear models (linear regression, logistic regression and Poisson regression) were used frequently in Eastern, Southern and Western Africa as compared to other analytical approaches, but to a varying degree. This can be attributed to the simplistic, flexible and intuitive way this approach accommodates predictors [84]. On the other hand, in the only study we identified that was conducted in Central Africa, a multivariate analysis was carried out. This further illustrates the relevance of these approaches in evaluating the relationships between georeferenced environmental variables and prevalence data, identifying potential risk factor(s)/predictor variable(s), explaining the observed variable(s) and forecasting prevalence at unsampled locations. The reliability of predictive geostatistical models formulated from a multivariate regression analysis is important in malaria mapping, and it depends on the selected variables fitted in the model. Researchers intending to employ either of the above-mentioned approaches should bear in mind that they do not intrinsically consider correlation in the errors [85]. Erroneous serial autocorrelation is likely to result in underestimated standard errors, and in addition, the evaluation of the impact of predictors would be biased. To account for the impact of autocorrelation on estimates, de Jong and Davidson [86] exhibited the relevance of applying heteroscedasticity and autocorrelation consistent estimators. A variety of statistical approaches have been applied across SSA to varying degrees and settings. The exploration and comparison of different statistical approaches and models for a particular setting would be useful in identifying and evaluating prediction accuracy. It will also be useful in identifying approaches that would provide accurate and reliable predictors for either short, long or intermediate prediction [87]. 

Overall, the quantitative models employed across localities and countries in SSA consistently revealed variability in the relationship between malaria and climatic/environmental variables. However, NDVI was observed to be the most significant predictor of malaria transmission followed by LST and RFE, and thus, they constituted the RS variable(s) that provided the best-fit model. To improve the overall predictive power and model robustness, we recommended the following: (1) Large datasets should be used over longer periods. For example, Nygren *et al.* [26] generated predictive models employing 126 weeks of data. Therefore, it will be difficult to know if the identified RS predictors of malaria transmission and the relationships they found will be sustained over time. (2) The incremental validity approach, which involves incorporating variables as supplemental to an identified predictor, should be practiced, as it can improve the predictive power [88]. For example, the study conducted by Ceccato *et al.* [43] revealed that NDVI predicted about 1%–20% of the variance in the southern and southeastern areas of Eritrea. This means that other RS-derived variables can explain 80%–99% of the variance. However, the addition of other RS-derived variables would be dependent on whether they improve the predictive validity of what the identified predictor predicts. (3) Linear models have been widely used across SSA. However, these models can result in inappropriate static regression and impose unrealistic or general assumptions. Thus, Bayesian models, which provide extensions of generalized linear models and are formulated to overcome some of the setbacks of linear models, should be employed.

The reviewed studies have shown that RS technology can contribute to the understanding of the complex nature of malaria across SSA. It can provide the potential climatic and environmental variables needed to identify significant spatially-explicit variable(s) associated with malaria risk and transmission. In areas like the Horn of Africa and Kenya, where malaria is highly seasonal, unstable and epidemic, the process of deciding climatic monitoring targets should be handled with caution to avoid the generation of unreliable malaria transmission models. Therefore, we are in support of regular capacity building and multidisciplinary collaboration between relevant departments, e.g., ecology, geography, biological science, epidemiology, entomology, information technology, statisticians, mathematical modelers, public health decision makers and stakeholders, in generating reliable prediction models. Furthermore, although this study can serve as an informative tool for public and environmental health workers, as well as researchers aiming to model potential climatic factors related to malaria and to delineate climate monitoring targets in SSA, some limitations should be noted. Firstly, relevant reports published in languages other than English and/or unpublished reports were excluded from this review. Secondly, studies that used only entomological data were also excluded.

## 5. Conclusions 

We conclude that RS technology is a vital tool in determining malaria risk predictors at regional, national and local scales in diverse regions of SSA. Our review suggests that the utilization of RS in determining reliable malaria transmission predictors and developing environmental monitoring would require a tailored approach that takes into account the geographical/climatic setting, the stage of the malaria elimination continuum, the characteristics of the RS variables and the analytical approach, which in turn, would support the channeling of intervention resources sustainably. The improvement of this technology has encouraged the acquisition and evaluation of a wide array of historical climatic/environmental variables at different spatial and temporal resolutions depending on the setting and intended usage. This therefore makes RS a relevant tool for identifying reliable climate-related malaria predictors that can be incorporated into an integrated malaria early-warning system or prediction model. Previously unidentified remotely-sensed variables, such as ETa and DWP, were found to be malaria transmission predictors, and EVI was also noted to be a suitable substitute for NDVI in denser vegetation, which needs to be further explored extensively across relevant localities and regions of SSA. Furthermore, the assessment of different statistical methods and models for a particular location would be useful in identifying and evaluating prediction accuracy depending on the length of prediction. The application of this technology can be further harnessed in generating reliable prediction models by devising means by which relevant skills and training and the easy acquisition of relevant RS-derived variables can be achieved. Therefore, relevant multidisciplinary collaborations, symposiums and capacity development are encouraged. 

## Figures and Tables

**Table 1:** Overview of studies that used RS-derived climatic variables and malaria epidemiological data in East Africa.

Reference(s)	Study Area(s)	Malaria Epidemiological Data	Climatic/Environmental Data Gotten via RS Technology		Environmental/Climatic Data from Other Sources	Statistical Method(s)	Main Findings
			Climatic/Environmental Data	Source(s) of RS Data			
[44]	Kenya: Western Kenya	Monthly inpatient confirmed cases	Multivariate El Nino Southern Oscillation Index (MENSOI)	NOAA	Monthly rainfall, mean monthly temperature	Time-series technique of spectral density analysis (SDA)	MENSOI did not influence teleconnection with monthly malaria incidence.
[39]	Kenya	Historic malaria distribution maps	NDVI, MIR, LST	NOAA-AVH R, Meteosat, USGS-DEM	-	Temporal Fourier analysis (TFA), discriminant analysis	LST was noted to be the best predictor of malaria transmission intensity. NDVI and CCD were identified as secondary predictors of transmission intensity. Altitude significantly improved the predictions.
	Uganda		CCD,				
	Tanzania		altitude				
[45]	Kenya: Kisii Central, Gucha, Nandi, and Kericho	Malaria cases (outpatients)	RFE	USGS	Seasonal climate forecast	WHO quartile, Cullen and cumulative sum (C-SUM) epidemic detection methods	Rainfall was able to forecast an epidemic one month in advance, but the outcome of seasonal climate forecast was erroneous and unreliable.
[24]	Kenya: Kisii Central, Gucha, Nandi, and Kericho	Malaria cases (outpatients)	RFE	USGS	Seasonal climate forecast	WHO quartile, Cullen and C-SUM epidemic detection methods	Seasonal climate forecasts did not predict the heavy rainfall. Rainfall estimates gave timely and reliable early warning, but monthly surveillance of malaria cases gave no effective warning.
[38]	Kenya	Malaria cases (outpatients)	Maximum temperature, minimum temperature and monthly rainfall	National Climate data Centre, NOAA	-	*t*-test, WHO Cullen epidemic detection methods, forward stepwise regression	Malaria incidence was significantly associated with monthly rainfall and maximum and minimum temperature at a time lag of 1–2 and 2–5 months, respectively.
	Ethiopia						
	Uganda						
[40]	Kenya Uganda Tanzania	Malariometric data from Mapping Malaria Risk in Africa (MARA/ARMA) (children between 0 and 15 years)	NDVI, MIR, LST CCD, altitude, land cover	NOAA-AVHRR, Meteosat, USGS-DEM, Landsat TM		TFA, discriminant analysis	NDVI, CCD and water body area were associated with malaria in the dry Ecozone 1. In Ecozone 2 where it was assumed that water was not generally limiting, LST and MIR were most abundant among the predictor variables selected.
[43]	Eritrea	Monthly clinical malaria cases	RFE, NDVI	CMAP, NOAA-AVHRR	Interpolated rainfall gauge data	Spearman and Pearson rank correlations, principal component analysis (PCA), non-hierarchical clustering analysis.	NDVI anomalies were highly correlated with malaria incidence anomalies, particularly in the semi-arid north of the country and along the northern Red Sea coast, which is a highly epidemic-prone area. CMAP rainfall correlated with malaria incidence anomalies, with a lead time of 2–3 months; while weather station rainfall correlated with malaria anomalies with a lag of 2 months.
[42]	Burundi: Karuzi	Monthly inpatient confirmed and unconfirmed cases	NDVI	AVHRR-NOAA	Rainfall, minimum and maximum temperature	ARIMA	NDVI, rainfall, mean maximum temperature and number of cases constituted the formation of the best predicting model (*R*^2^_adj_ = 82%, *p* < 0.0001 and 93% forecasting accuracy in the range ±4 cases per 100 inhabitants). NDVI, rainfall and maximum temperature were noted to correlate with malaria cases.
[25]	Eritrea	Monthly clinical malaria cases	RFE, NDVI	CMAP NOAA-AVHRR		Regression analysis	The Poisson regression analysis showed that CMAP rainfall estimates were significantly associated with malaria with a lead time of 2–3 months in Gash Barka. NDVI showed a similar relationship in Anseba.
[46]	Somalia	Survey of *P. falciparum* parasite rate (PfPR)	EVI	MODIS	Precipitation, temperature, distance to permanent water bodies	Logistic regression models, kriging, Bayesian binomial generalized linear geostatistical models	The non-spatial bivariate logistic regression analysis showed that EVI, precipitation, maximum and minimum temperature and distance to water were highly significantly associated with PfPR. After employing the above covariates in the multivariate Bayesian geostatistical model, only temperature and precipitation remained significant (odds 95% confidence interval (CI)) at the southern part of Somalia.
[47]	Kenya: Nandi and Kisii	Confirmed and unconfirmed, monthly inpatient and outpatient cases	Dipole mode index (DMI), El Nino-Southern Oscillation (ENSO) index Nino 3 region (NINO3)	NOAA	Rainfall	Time series regression, Poisson generalized linear model (GLM), Pearson’s correlation	No strong association was found between NINO3 and the number of malaria cases after adjusting for the effect of DMI. Malaria cases increased by 3.4%–17.9% for each 0.1 increase above a DMI threshold value lagged at 3–4 months. Malaria cases increased by 1.4%–10.7% for each 10-mm increase in monthly rainfall lagged at 1–3 months.
[48]	Tanzania	Survey of confirmed malaria cases among children less than 5 years old	LST, NDVI, altitude	MODIS DEM-USGS	Rainfall, permanent water bodies	Multivariate logistic regression, Bayesian kriging	The bivariate analyses showed that altitude was negatively associated with malaria risk at the 5% significance level, indicating that children at above 1500 m had a lower risk of malaria. Rainfall, NDVI, day and night LST were positively associated with parasitemia risk.
[20]	Ethiopia: Amhara region	Monthly confirmed outpatients cases	LST, NDVI, enhanced vegetation index (EVI), actual evapotranspiration (ETa), RFE	MODIS TRMM, NASA, and the Japan Aerospace Exploration Agency (JAXA)		Seasonal autoregressive integrated moving average (SARIMA)	RFE, EVI, LST and ETa served as suitable malaria predictor as they improved the model fit, and they revealed a lagged positive association with malaria cases. ETa, which was utilized in malaria epidemiological study for the first time, showed a significant positive correlation with malaria at lags from 1–3 months in 3 of the 12 sites studied. EVI had a 3-month lag at 3 sites, while rainfall lagged by 1–3 months at 5 sites. LST exhibited a positive association lagged by 1–6 month at 6 sites.
[49]	Somalia	Survey of PfPR data among children of 2 to less than 10 years	EVI	MODIS	Annual mean precipitation, temperature suitability index (TSI), distance to larva breeding sites.	Linear regression, Space-time model-based geostatistical (MBG) method	The inclusion of 1 km^2^ MODIS EVI (odds ratio (OR) = 0.81, 95% CI = 0.19–1.44, *p*-value = 0.011) and other covariates (precipitation, floodplains, distance to main water bodies) in the analysis served as the best predictor for PfPR.

**Table 2:** Overview of studies that used RS-derived climatic variables and malaria epidemiological data in Southern Africa.

Reference(s)	Study Area(s)	Malaria Epidemiological Data	Climatic/Environmental Data Obtained via RS Technology		Environmental/Climatic Data from Other Sources	Statistical Method(s)	Main Findings
			Climatic/Environmental Data	Source(s) of RS Data			
[50]	Zimbabwe	Monthly confirmed and unconfirmed cases (children less than 5 years old)	NDVI	NOAA-AVHRR	Rainfall, maximum temperature, minimum temperature, vapor pressure	Bayesian Poisson model	Vapor pressure, rainfall, mean monthly (28–32 °C) and maximum temperature (24–28 °C), showed a significant positive correlation with malaria incidence, while NDVI, high monthly maximum and minimum temperatures showed a negative association.
[51]	Botswana	Confirmed malaria incidence data	RFE, sea surface temperature (SST)	CMAP		Stepwise regression, Spearman’s rank order, Pearson’s product moment correlation, quadratic test, logistic regression, Mann–Whitney U-tests	Negative anomalies of December–January SSTs were significantly associated with December–January rainfall estimates (Pearson’s *R* = −0.55 (−0.76 to −0.22) and Spearman’s *R* = −0.59 (−0.81 to −0.18)), as well as with the standardized malaria incidence anomalies and accounted for nearly 25% of the inter-annual variance in malaria incidence.
[52]	Zimbabwe	Annual confirmed and unconfirmed malaria case (children less than 5 years old)	NDVI	NOAA-AVHRR (NASA)	Rainfall, vapor pressure, mean temperature, maximum temperature, minimum temperature	Markham’s seasonality index, Negative binomial regression analysis, Bayesian negative binomial models	In the bivariate analysis NDVI, vapor pressure, rainfall, average monthly (28 °C–32 °C) and maximum (24 °C–29 °C) temperature range revealed a significant positive correlation (*p* < 0.001) with malaria incidence. After employing the spatiotemporal model, NDVI became insignificant.
[53]	Botswana	Confirmed malaria incidence data	RFE	CMAP	SST	Probabilistic prediction, Kolmogorov–Smirnov test, quadratic test	Higher than expected malaria years were associated with above-average rainfall, while the lowest malaria years were associated with below average rainfall.
[54]	Botswana	Malaria prevalence data (children between 1 and 14 years age)	NDVI, RFE	NOAA-AVHRR, CMAP	Elevation, surface water land cover, temperature vapor pressure	Univariate logistic regression analysis, stepwise bootstrap method	RFE (OR = 2.01, 95% CI = 1.47–2.70), annual mean temperature (OR = 5.75, 95% CI = 4.14–8.08) and elevation (OR = 1.82, 95% CI = 1.49–2.22) were significantly associated with malaria prevalence after allowing for spatial correlation.
[28]	Angola	Survey of confirmed malaria cases (children less than 5 years old)	Day LST, night LST, NDVI, altitude	MODIS, USGS-DEM	Rainfall	Bayesian logistic regression, Bayesian kriging	NDVI (95% CI = 6.28, 17.94; OR = 10.62) and rainfall (95% CI = 6.00, 19.43; OR = 10.80) showed a significantly positive relationship with malaria incidence after carrying out a bivariate analysis.
[55]	Zambia	Survey of confirmed malaria cases among children less than 5 years old	Day LST, night LST, NDVI, land cover, altitude	MODIS, USGS-DEM	RFE, water bodies (lakes, rivers and wetlands)	Lag time analysis, bivariate and multiple geostatistical logistic regression analysis, Bayesian kriging	NDVI, night LST at 1-km^2^ spatial resolution and rainfall within the last 2.7 months showed positive significant association, while day LST reflected a significant negative relationship.
[56]	Namibia: Northern Namibia	Monthly confirmed malaria cases	EVI, precipitation	MODIS, TRMM-NASA and JAXA	Temperature suitability index (TSI)	Non-spatial Poisson regression, Bayesian spatio-temporal zero-inflated conditional autoregressive (CAR) model, zero-Inflated Poisson (ZIP) model	Initially, the univariate non-spatial regression analysis indicated that the EVI (coefficient of regression, 95% CI: 6.55, 4.25–8.87, *p* < 0.001), the temperature suitability index acquired from the Malaria Atlas project (7.57, 5.34–9.96, *p* < 0.001) and precipitation (0.02, 0.01–0.03, *p* = 0.002) were significant predictors. However, after employing the best performing predictive model (the multivariate model), only EVI (coefficient of regression, 95% CI: 14.29, 9.24–19.42, *p* < 0.001) was positively correlated.
[57]	Swaziland	Monthly confirmed malaria cases (imported and locally-acquired)	NDVI, NDWI, elevation, TWI	Landsat-7 ETM+, SRTM	Temperature, rainfall, distance to nearest water body	Satterthwaite *t*-tests, logistic regression mixed model, random forest	Case households during the high transmission season tended to be located in areas of lower elevation, closer to bodies of water, in more sparsely-populated areas, with lower rainfall and warmer temperatures and closer to imported cases than random background points (all *p* < 0.001). In relation to model accuracy, NDWI was the most important RS-derived variable followed by NDVI and, lastly, TWI.
[58]	Malawi	Monthly confirmed and unconfirmed cases	Precipitation, altitude	NOAA Climate Prediction Centre SRTM	temperature	Negative binomial generalized linear model (GLM), generalized linear mixed model (GLMM), Kernel density	The negative binomial with only fixed effects was used to determine the best time lags between climatic variables and malaria. It showed that at the 0.05 significance level, precipitation and temperature were statistically significant at Lag 1–3. The maximum relative malaria risk is observed to be the maximum temperature of 28 °C and precipitation of 6.24 mm·day^−1^.
[26]	Zambia: Southern Province	Weekly confirmed malaria cases	Rainfall, NDVI, DWP, LST, elevation	TAMSAT, MODIS, ASTER	-	Kruskal-Wallis tests, Ljung–Box Q statistics, Kriging, ARIMAX	NDVI, DWP and night LST were the highly significant predictors at the high and low malaria transmission malaria zones partitioned in the study area.

**Table 3:** Overview of studies that used RS-derived climatic variables and malaria epidemiological data in West Africa.

Reference(s)	Study Area(s)	Malaria Epidemiological Data	Climatic/Environmental Data Gotten via RS Technology		Environmental/Climatic Data from Other Sources	Statistical Method(s)	Main Findings
			Climatic/Environmental Data	Source(s) of RS Data			
[59]	Mali	Malaria prevalence data extracted from the MARA/ARMA database	NDVI	NOAA-AVHRR	Rainfall, average maximum temperature, average minimum temperature, distance to the nearest water body	Logistic regression analysis, kriging	Mean NDVI from June–November (wet season), mean maximum temperature from March–May, months with more than 60 mm of rainfall and distance to water bodies were the significant independent variables for predicting malaria prevalence.
[60]	Mali	Malaria prevalence data extracted from the MARA/ARMA database	NDVI	NOAA/NASA-AVHRR	Temperature, duration of rainy season, distance to water	Garki mode, Bayesian models and kriging	During the raining season, NDVI and temperature had no statistical relationship with entomological inoculation rate (EIR). Distance to water was significantly related to transmission intensity, indicating high transmission in the areas within 4 km of the water source.
[27]	Mali	Malaria prevalence data from the MARA/ARMA database (children between 1 and 10 years old)	NDVI	NASA-AVHRR	Temperature, rainfall, water bodies, season length	Bayesian logistic regression, Bayesian non-stationary model, Bayesian kriging	The non-stationary model showed that NDVI and minimum temperature had a positive statistical relationship with malaria risk, awhile rainfall had a negative statistical relationship.
[61]	Côte d’Ivoire: Man	Confirmed *P. falciparum* survey in children between 6 and 16 years	NDVI, LST, RFE	MODIS-USGS Meteosat 7	Distance to the nearest river	Bivariate logistic regression models	In bivariate non-spatial models, NDVI, RFE and distance to rivers, were significantly associated with a *P. falciparum* infection. However, after employing the spatial correlation, NDVI showed only a ‘borderline’ significance with *P. falciparum* prevalence.
[23]	Mali: Bancoumana	Confirmed *P. falciparum* survey in children between 0 and 12 years	NDVI	NOAA-AVHRR		ARIMA	The seasonal analytical approach revealed that the seasonality of *P. falciparum* incidence was significantly explained by NDVI with s 15-day lag (*p* = 0.001). The NDVI threshold was 0.361 (*p* = 0.007).
[6]	West Africa	MARA/ARMA Malaria prevalence date among children between 1 and 10 years	NDVI, land use	NOAA-AVHRR USGS	Temperature, rainfall, soil water storage index (SWS), water bodies, agro-ecological zones	Logistic regression model, non-parametric regression models	NDVI was not associated with malaria in any of the four defined agro-ecological zones (Equatorial forest, Guinea savannah, Sahel region, Sudanese savannah).
[62]	Côte d’Ivoire: Man	Survey of confirmed malaria cases among school children of Grades 3–5	NDVI, LST, RFE DEM	MODIS-USGS Meteosat 7 SRTM		Bayesian negative binomial regression models, Bayesian kriging	The bivariate non-spatial analysis identified NDVI, RFE, LST and close proximity to standing water (rivers, swamps and irrigated fields) as significant risk malaria factors. After employing the spatial analyses, only mean RFE remained significant over the malaria transmission season (June–August).
[63]	Senegal	Survey of confirmed malaria cases among children less than 5 years old	Day LST, night LST, NDVI, altitude	MODIS USGS-DEM	Rainfall, permanent rivers and lakes	Bayesian geostatistical zero-inflated binomial (ZIB), Bayesian kriging	Night LST (OR 1.16; 95% CI (0.66, 1.86)) and NDVI (OR 1.48; 95% CI (0.88, 2.48)) were noted to have a positive association with malaria parasitemia.
[64]	Côte d’Ivoire	Malaria prevalence data for children aged less than 16 years	LST, NDVI Elevation	MODIS, USGS-DEM	Rainfall, distance to the nearest water body	Binomial regression models, Bayesian non-spatial and geo-statistical logistic regression models, Bayesian kriging	In the non-stationary spatial model (the best model), the covariates rainfall (OR = 0.76; Bayesian credible interval (BCI) = 0.70, 0.83) and maximum LST (OR = 0.72; BCI = 0.64, 0.79) were significantly negatively associated with Plasmodium prevalence.

**Table 4:** Overview of a study that used RS-derived climatic variables and malaria epidemiological data covering Central and Western Africa.

Reference(s)	Study Area(s)	Malaria Epidemiological Data	Climatic/Environmental Data Gotten via RS Technology		Environmental/Climatic Data from Other Sources	Statistical Method(s)	Main Findings
			Climatic/Environmental Data	Source(s) of RS Data			
[65]	West Africa and Central Africa	Malaria prevalence data extracted from the MARA/ARMA database	NDVI, land use	NASA-AVHRR USGS-NASA	Temperature, rainfall, soil water storage index, water bodies, agro-ecological zones, transmission seasonality	Multivariate analysis, Garki model, Bayesian linear geostatistical model, Bayesian kriging	NDVI, distance from water, length of season, rainfall and maximum temperature correlated significantly with malaria transmission intensity and were included in the best fitting model. NDVI had a significant positive association with malaria transmission, except for areas distant from water bodies. This negative association between malaria transmission and distance to water was observed in regions with NDVI values greater than 0.6.

**Table 5:** Commonly-used RS variables in SSA.

RS Variables	Description	Sources
NDVI	This is an indicator of the greenness of the biomass and varies between −1 and +1. It is calculated as [66,67]: $\frac{\left(\text{NIR}-\text{Red}\right)}{\left(\text{NIR}+\;\text{Red}\right)}$	MODIS, NOAA-AVHRR
LST (day and night)	This can be estimated from thermal infrared sensors. It is sensitive to the thermal characteristics of the ground and atmospheric effects of spectral radiation [68].	MODIS, NOAA-AVHRR
RFE/CCD	This provides indirect estimates of rainfall based on the detection of precipitation particles or the duration a cloud top is below a threshold temperature [69].	TRMM, CMAP, Meteosat
EVI	EVI provides an alternative to NDVI because it improves sensitivity over areas of denser vegetation. It is calculated as [66]: $G\;\frac{\left(\text{NIR}-\text{Red}\right)}{\left(\text{NIR}+C1\;\times \text{Red}-C2\times \text{Blue}+L\right)}$, where *G* is a gain factor, *C*1 and *C*2 are aerosol resistance coefficients and *L* is the canopy background adjustment that addresses nonlinear, differential NIR and red radiant transfer through a canopy.	MODIS
Elevation/altitude	This correlates negatively with temperature and positively with precipitation and can be applied as a surrogate indicator [69].	USGS-DEM, ASTER, SRTM
Land use and land cover	This is related to the natural and physical environment and the human activities on the landscape [66].	MODIS, Landsat TM, USGS-NASA

**Table 6:** Overview of the RS satellites/sensors used in the malaria epidemiological studies in SSA.

Satellite/Sensors	Spectral Range	Spatial Resolution	Revisit Time	Swath Width	Radiometric Resolution
NOAA/NASA-AVHRR	0.58–12.50 µm	1.1 km	12 h	2900 km	10 bit
MODIS	0.40–14.50 µm	250 m, 500 m, 1 km	1–2 days	2330 km	12 bit
Landsat TM ^1^	0.45–12.5 µm	30 m, 120 m	16 days	185 km	8 bit
Landsat-7 ETM+ ^2^	0.45–12.5 µm	15 m, 30 m, 60 m	16 days	185 km	9 bit (8 bit transmitted)
Meteosat 1–7	0.50–12.5 µm	2.5 km, 5 km	30 min	-	8 bit
Meteosat 8–10	0.40–14.40 µm	1 km, 3 km	15 min		10 bit
TRMM	VIRS ^3^: 0.63 µm, 1.60 µm, 3.75 µm, 10.7 µm, and 12 µm	VIRS: 2 km TMI ^4^: 5–45 km PR ^5^: 4.3 km	3 hourly, daily, monthly	VIRS: 720 km TMI: 780 km PR: 215 km	-
SRTM	-	30 m	16 times per day	C-radar: 225 km X-radar: 50 km	C-radar: 8 bit X-radar: 6 bit
ASTER	VNIR ^6^: 0.52–0.86 µm SWIR ^7^: 1.60–2.43 µm TIR ^8^: 8.125–11.65 µm	VNIR: 15 m SWIR: 30 m TIR: 90 m	5 days 16 days 16 days	60 km 60 km 60 km	VNIR: 8 bit SWIR: 8 bit TIR: 12 bit
CMAP	-	0.25° × 0.25°	5 days, monthly	-	-

^1^ Thematic Mapper; ^2^ Enhanced Thematic Mapper plus; ^3^ Visible Infrared Scanner; ^4^ TRMM Microwave Imager; ^5^ Precipitation Radar; ^6^ Visible Near Infrared; ^7^ Shortwave Infrared; ^8^ Thermal Infrared.

**Table 7:** Overview of new generation RS satellites/sensors with improved characteristics for malaria modelling.

Satellite/Sensors	Spectral Range	Spatial Resolution	Revisit Time	Swath Width	Radiometric Resolution
Landsat-8	0.43–12.5 µm	15 m, 30 m, 100 m	16 days	185 km	12 bit
Copernicus: Sentinel-2	0.43–2.28 µm	10 m, 20 m, 60 m	5 days	290 km	12 bit
GPM	-	250 m, 500 m	3 h	120 km, 245 km, 885 km	-
SMAP	-	3 km, 10 km, 40 km	2 days, 3 days	1000 km	-
SPOT 6 and SPOT 7	0.45–0.89 µm	1.5 m, 2 m, 6 m, 8 m	1–5 days	60 km	12 bit

## Abbreviations

The following abbreviations are used in this manuscript:

SSA	Sub-Saharan Africa
RS	remote sensing
ISI	Institute for Scientific Information
NDVI	normalized difference vegetation index
NOAA	National Oceanic and Atmospheric Administration
AVHRR	Advanced Very High Resolution Radiometer
MODIS	Moderate-resolution Imaging Spectrometer
WHO	World Health Organisation
ARIMA	autoregressive integrated moving average
RFE	rainfall estimates
CMAP	Climate Prediction Centre Merged Analysis of Precipitation
LST	land surface temperature
USGS	United States Geological Service
DEM	digital elevation model
ADDS	Africa Data Dissemination Service
MIR	mid-infrared
CCD	cold cloud duration
ETa	actual evapotranspiration
EVI	enhanced vegetation index
MARA/ARMA	Mapping Malaria Risk in Africa
NDWI	normalized difference water index
TWI	topographic wetness index
ETM+	Enhanced Thematic Mapper plus
SRTM	Shuttle radar Topography Mission
DWP	nocturnal dew point
TAMSAT	Tropical Application of Meteorology using satellite data and ground-based observations
GPM	Global Precipitation Measurement mission
SMAP	Soil Moisture Active/Passive mission
